# Bortezomib enhances the anti-cancer effect of the novel Bruton’s tyrosine kinase inhibitor (BGB-3111) in mantle cell lymphoma expressing BTK

**DOI:** 10.18632/aging.203314

**Published:** 2021-09-10

**Authors:** Xianhuo Wang, Yue Fei, Xia Liu, Tingting Zhang, Wei Li, Xiaohui Jia, Xianming Liu, Lihua Qiu, Zhengzi Qian, Shiyong Zhou, Xiubao Ren, Qiongli Zhai, Bin Meng, Lanfang Li, Huilai Zhang

**Affiliations:** 1 Department of Lymphoma, Tianjin Medical University Cancer Institute and Hospital, National Clinical Research Center of Cancer, Key Laboratory of Cancer Prevention and Therapy, Tianjin’s Clinical Research Center for Cancer, The Sino-US Center for Lymphoma and Leukemia Research, Tianjin 300060, China; 2 Department of Biotherapy, Tianjin Medical University Cancer Institute and Hospital, Tianjin 300060, China; 3 Department of Pathology, Tianjin Medical University Cancer Institute and Hospital, Tianjin 300060, China

**Keywords:** BGB-3111, Bruton’s tyrosine kinase, bortezomib, mantle cell lymphoma

## Abstract

BGB-3111, a novel Bruton’s tyrosine kinase (BTK) inhibitor, shows promising anti-cancer effects in chronic lymphocytic leukemia/small lymphocytic lymphoma (CLL/SLL), mantle cell lymphoma (MCL), and Waldenstrom macroglobulinemia (WM). This study aimed to investigate the anti-cancer effects of BGB-3111 combined with bortezomib (BTZ) against the BTK-expressing MCL. We found that BTK, which was overexpressed in 59.4% of patients with MCL, was mainly characterized by high Ki67 and elevated MIPI scores. BGB-3111 strongly inhibited cell proliferation, induced cell cycle arrest in the G1/G0-phase, and promoted cell apoptosis in the MCL cells expressing BTK. BGB-3111 provides better safety than another BTK inhibitor, ibrutinib as ibrutinib inhibits the inducible T-cell kinase (ITK) as an off-target effect but BGB-3111 does not inhibit ITK. Low doses of BTZ enhanced the anti-cancer effect induced by the low dose of BGB-3111 by downregulating the expression levels of PARP and Bcl-2 and increasing the expression levels of cleaved PARP and cleaved caspase-9. In addition, low doses of BGB-3111, but not of BTZ, inhibited BTK phosphorylation. However, low-doses of BTZ strengthened the anti-cancer effect induced by the low-doses of BGB-3111 via synergistically suppressing the IκBα and P65 phosphorylation. Taken together, our findings validate that BGB-3111 is a novel and effective BTK inhibitor for MCL-expressing BTK. Hence, it can be harnessed as a potential therapeutic strategy through a combinatorial treatment comprising low-dose BGB-3111 and low-dose BTZ to gain strong anti-cancer effects and better safety for MCL patients.

## INTRODUCTION

Mantle cell lymphoma (MCL) originates from B lymphocytes in the region of the small lymph node, which accounts for 3% to 10% of non-Hodgkin’s lymphoma (NHL) [1–5]. A majority of MCL patients exhibit an aggressive disease with a poor prognosis [6, 7]; however, a subset of patients may initially exhibit an indolent disease course [8–12]. The genetic characteristics of MCL include t(11;14) (q13;q32) translocation and cyclin D1 (CCND1) overexpression, which usually causes deregulation of the cell cycle at the G1-S phase transition [13]. Although this translocation is considered to play a primary role in MCL pathogenesis, other carcinogenic events are also required for MCL progression [14–17]. In addition to its pathogenic features, the tissue microenvironment is critical to support MCL cell growth [18].

MCL lymphoma cells are derived from pre-germinal center B-cells and are usually activated by growth factors, which can bind to cell surface receptors. The B-cell receptor (BCR) signaling is crucial for normal B-cell development. It regulates multiple biological processes, including cell proliferation, differentiation, apoptosis, and migration [15, 19]. Bruton’s tyrosine kinase (BTK) has been identified as a key component of BCR signaling, which can activate the downstream NF-κB pathway [20]. Ibrutinib is a first-generation BTK inhibitor that can inhibit BTK irreversibly and non-specifically bind to Cys-481 [19]. Ibrutinib has also become a new standard of care for the treatment of patients with relapsed or refractory chronic lymphocytic leukemia/small lymphocytic lymphoma (CLL/SLL), MCL, and Waldenstrom macroglobulinemia (WM) [21]. However, atrial fibrillation (AF) [22, 23], ventricular arrhythmias [24], and bleeding [25] are emerging as potential threats limiting the desired potential effects. The ibrutinib-induced adverse events may be attributed partly to its off-target effects, involving other kinases, especially the Tec family proteins [26]. In addition, recent reports have demonstrated the possibility of ibrutinib resistance [27–31]. Therefore, it is necessary to develop highly specific BTK inhibitors for lymphoma treatment.

BGB-3111 is a novel and next-generation BTK inhibitor, which is designed to selectively target BTK with less off-target activities. Studies have reported that BGB-3111 is less potent in inhibiting rituximab-induced antibody-dependent cellular cytotoxicity compared to ibrutinib [32, 33]. Recent studies have also shown that BGB-3111 has a promising response rate in patients with CLL/SLL, MCL, and WM [34–38]. However, it is still necessary to further study the anti-cancer effects of BGB-3111 and the combination therapy strategies with low-dose BGB-3111 and other targeted agents, which can induce a strong anti-cancer effect with reduced toxicity in MCL.

The NF-κB signaling plays a key role in the pathogenesis of MCL. NF-κB regulates the transcription of genes essential for cell survival, proliferation, inflammation, and invasion/metastasis [39]. Bortezomib (BTZ) has been reported to be a reversible proteasome inhibitor, which could effectively block the activation of the NF-κB pathway. The development of bortezomib provided a transient response in inducing responses in the relapsed setting; however, these treatments provided moderate responses with toxicities, and the patients eventually recovered [40]. Several trials evaluating bortezomib in combination with other therapeutic agents have also been reported, but the results are disappointing because of the poor response rate and increased treatment-related adverse events [40–46]. A previous study suggested that the proteasome inhibitors suppress NF-κB activity by stabilizing the inhibitory molecule IκB, which binds NF-κB and prevents its nuclear translocation, thereby downregulating the levels of its targets and producing a potent antimyeloma effect [47]. Therefore, BTK inhibitors and proteasome inhibitors play crucial roles in blocking the activation of the NF-κB pathway in different ways. This demonstrated that there may be a synergistic effect between the BTK inhibitor and proteasome inhibitor. In this study, we investigated the anti-cancer effect of BGB-3111 and a combination therapy strategy, in which a low dose of BTZ enhanced the anti-cancer effect of BGB-3111 at a low dose in MCL.

## RESULTS

### BTK expression in tissues of MCL patients

**Figure 1:**
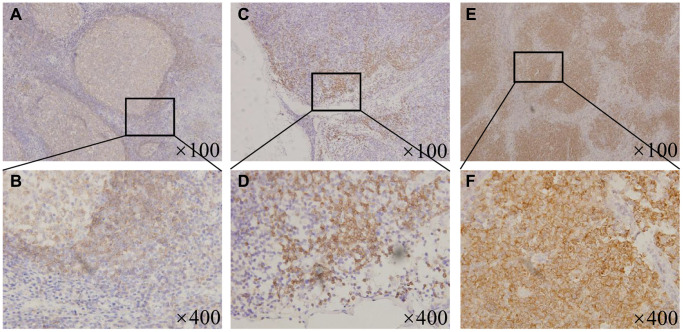
**BTK expression in the MCL patients and the benign lymphoid tissues.** (**A**) ×100 magnification and (**B**) ×400 magnification, Representative patterns of BTK expressing limitedly in the germinal center cells of the mantle zone in the tonsils. (**C**) ×100 magnification and (**D**) ×400 magnification, Representative patterns of low BTK expression with less than one-quarter positive staining proportion in the MCL patient tissues. (**E**) ×100 magnification and (**F**) ×400 magnification, Representative patterns of high BTK expression with widely broken and disappeared germinal center in the MCL patient tissues.

To explore the potential clinical value of BTK inhibitors in MCL patients, we first studied the expression levels of BTK. The baseline characteristics of all patients were as follows: the median age was 61 years (range: 42–75 years). Of the 32 patients, 23 (71.9%) were male, 9 (28.1%) were female, 29 (90.6%) were in the advance diseased stage, 23 (71.9%) were low middle, 9 (28.1%) were middle–high or high risk, 20 (62.5%) had bone marrow involvement, 16 (50%) had elevated lactate dehydrogenase (LDH) levels, and 14 (43.8%) had elevated beta 2 microglobulin (β2-M) levels. Supplementary Table 1 summarizes the association between the BTK expression and the clinicopathological parameters. Results showed that BTK expression was low in all the benign reactive hyperplasia of lymph nodes and was limited to the germinal center cells of the mantle zone (Figure 1A and 1B). Thirteen (40.6%) patients were categorized into the low BTK expression group, which was characterized by less than one-quarter positive staining proportion (Figure 1C and 1D), and 19 (59.4%) patients were categorized into the high BTK expression group, which was characteristic of a widely broken and disappeared germinal center (Figure 1E and 1F). The correlation analysis between the BTK expression and clinical characteristics revealed that the patients with higher BTK levels were always accompanied by high Ki67 (>30%, *P* = 0.019) and elevated MIPI scores (*P* = 0.038). Therefore, we found that BTK was overexpressed in the majority of the MCL patients with high Ki67 and MIPI scores, suggesting that the strategy of specifically inhibiting BTK has a promising clinical value for treating MCL.

### BGB-3111 inhibits the proliferation in MCL cells expressing BTK

**Figure 2:**
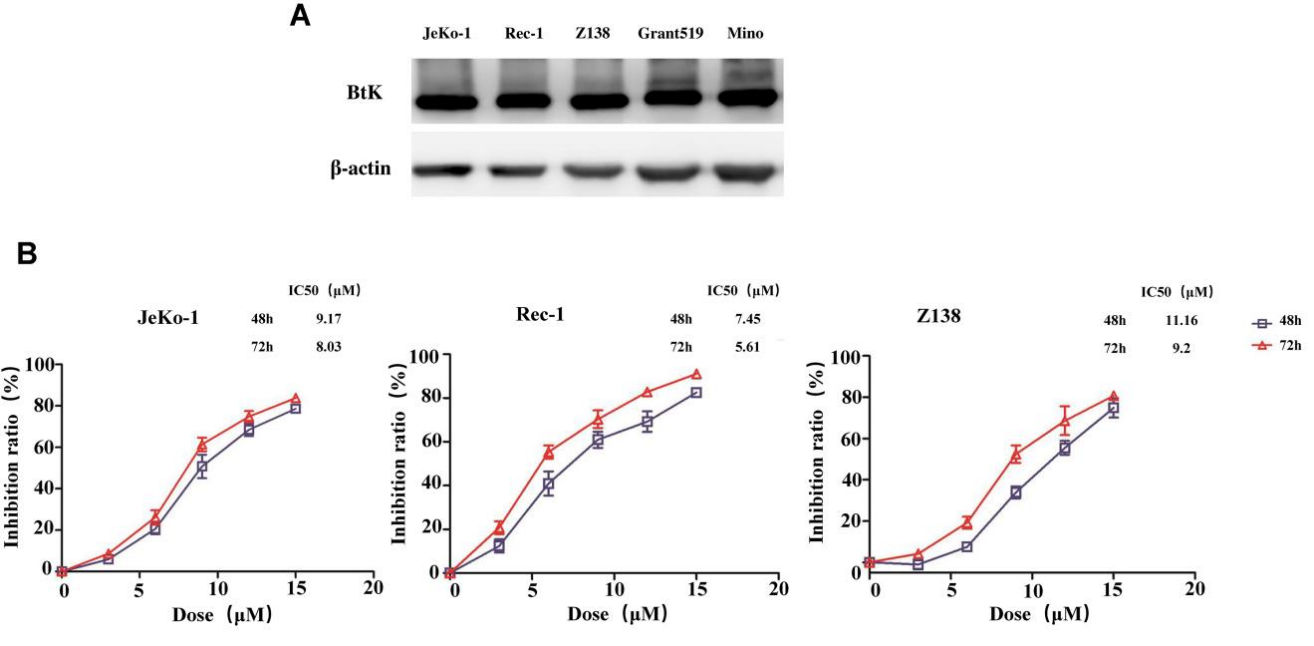
**BTK expression in the MCL cell lines and cell viability treated with BGB-3111.** (**A**) The BTK expression in the five MCL cell lines. (**B**) Cell viability in the Jeko-1, Rec-1, and Z138 treated with BGB-3111 for 48 or 72 h. Results are the mean ± SD of three independent experiments.

To evaluate the cell proliferative effect of BGB-3111, we first measured the BTK expression in MCL cell lines. The results revealed that five MCL cell lines had high BTK expression (Figure 2A), out of which three MCL cell lines, including Jeko-1, Rec-1, and Z138, were selected for further studies. Cell growth was detected in the three cell lines treated with increasing doses of BGB-3111 from 0 to 15 μM for 48 h or 72 h. Results showed that BGB-3111 inhibited the cell viability in a dose- and time-dependent manner (Figure 2B). The IC50 values were respectively 9.17 μM, 7.45 μM, and 11.16 μM after 48 h treatment, and were respectively 8.03 μM, 5.61 μM, and 9.2 μM after 72 h treatment for the Jeko-1, Rec-1, and Z138 cell lines. Our findings showed that BGB-3111 had a strong anti-cancer effect in the MCL cell lines expressing BTK. Furthermore, after 48 h treatment with BGB-3111, the subsequent experiments were performed.

### BGB-3111 ascends cell-cycle arrest and induces apoptosis in the MCL cells expressing BTK

**Figure 3:**
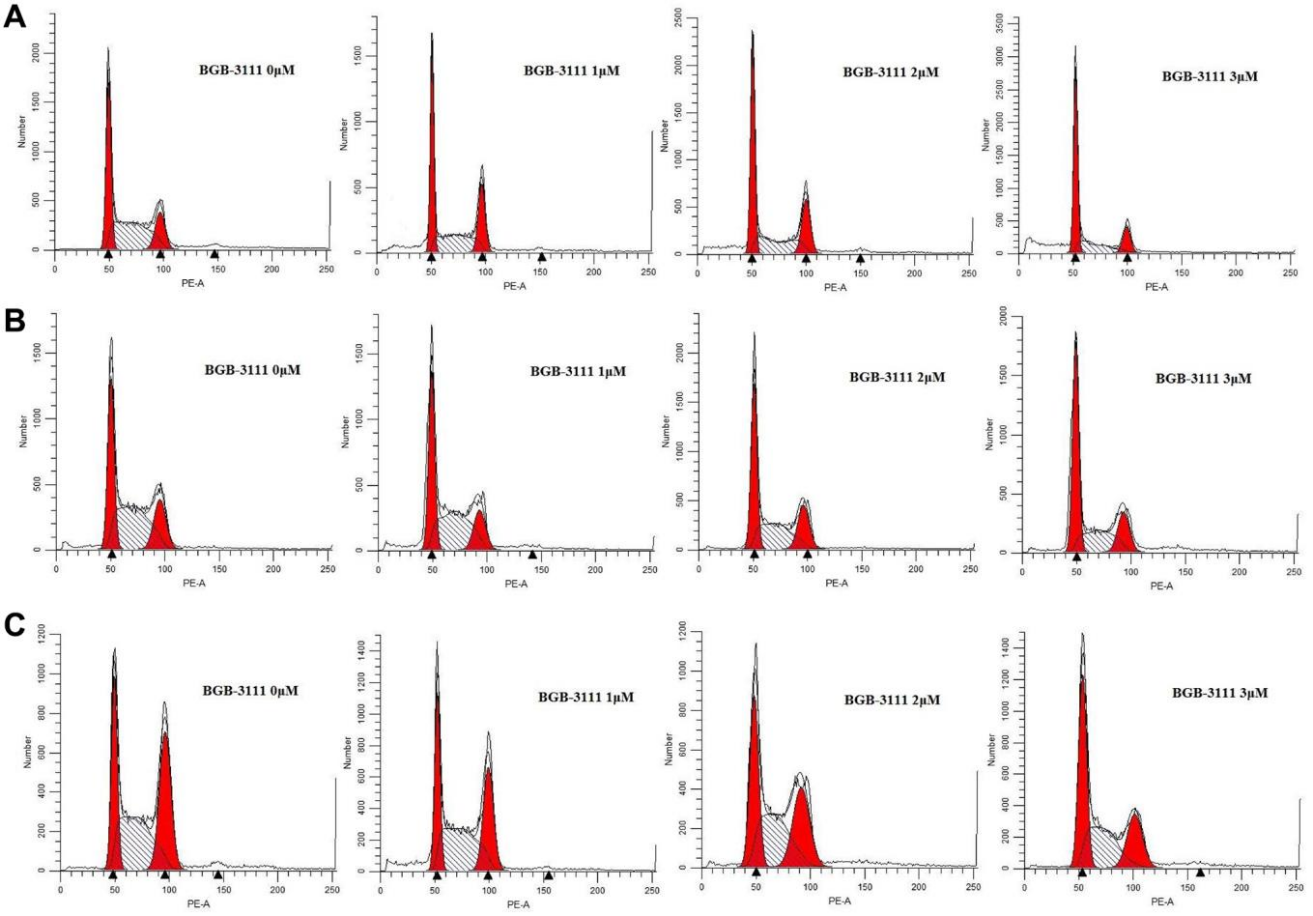
**Cell-cycles are arrested at G1/G0-phase in the MCL cell lines after being treated with BGB-3111.** Cell cycles of Jeko-1 (**A**) Rec-1 (**B**) and Z138 (**C**) treated with BGB-3111 at the doses of 0 μM, 1 μM, 2 μM, and 3 μM for 48 h. The cell percentage of the cell cycle was detected using flow cytometry.

Cell cycle analysis was performed to characterize the cytotoxicity of BGB-3111 in the Jeko-1, Rec-1, and Z138 cells. After treatment with BGB-3111 (0, 1, 2, or 3 μM) for 48 h, the cells were arrested in the G1/G0 phase in a dose-dependent manner, while the percentage of cells in the G2/M phase was significantly reduced (Figure 3 and Supplementary Figure 1). The percentage of G1/G0 phase cells changed from 33.4% ± 3.01% to 53.04% ± 6.42%, 30.85% ± 2.77% to 49.89% 2.13%, and 27.76% ± 1.53% to 42.34% ± 3.81% in the Jeko-1, Rec-1, and Z138 cells with the increasing doses of BGB-3111 treatment (*p* < 0.05). Together, our findings suggest that BGB-3111 could induce cell cycle arrest in the G1/G0-phase in the MCL cells.

**Figure 4:**
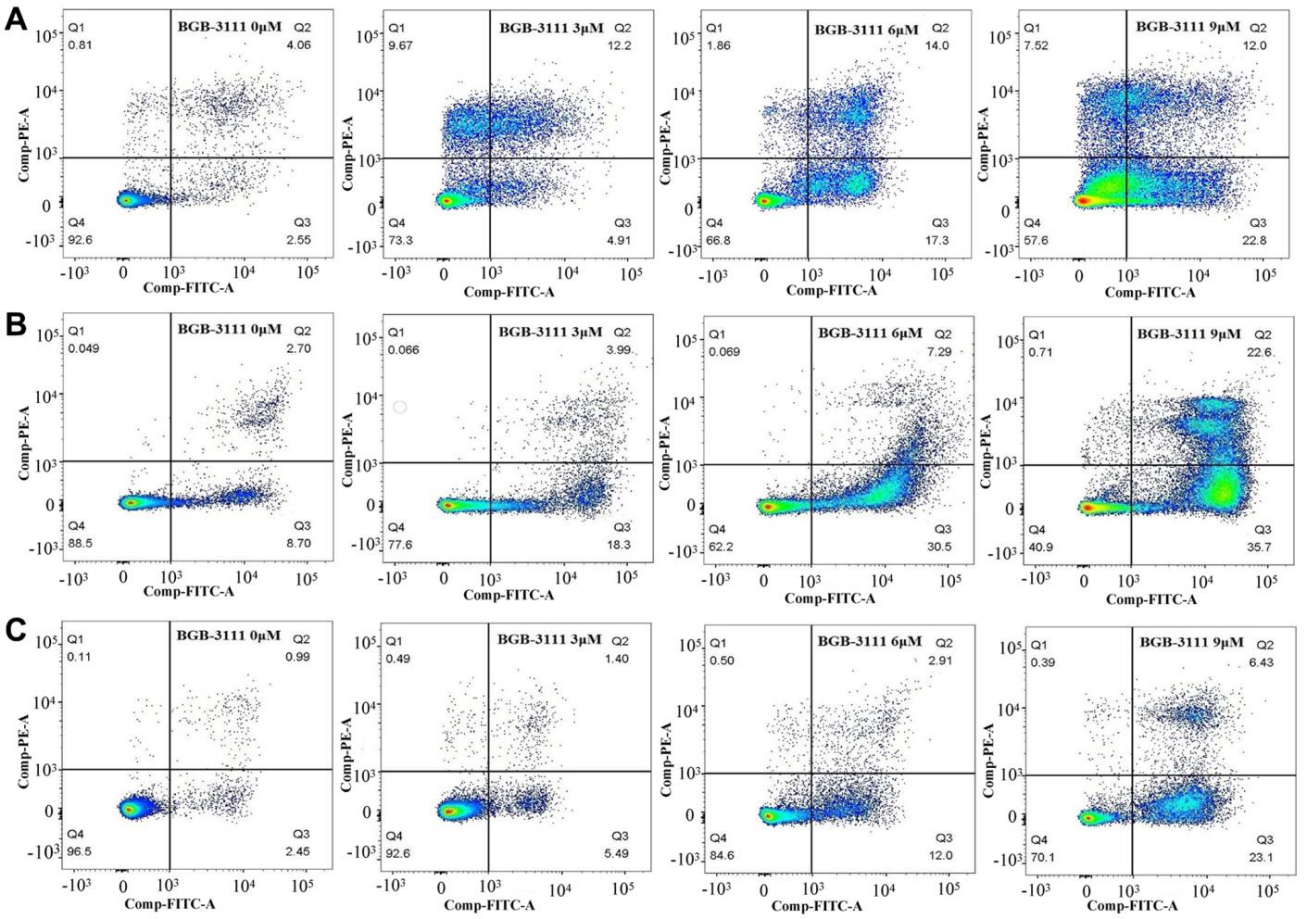
**Cell apoptosis in the MCL cell lines after being treated with BGB-3111.** The cell apoptosis of Jeko-1 (**A**) Rec-1 (**B**) and Z138 (**C**) treated with BGB-3111 at the doses of 0 μM, 3 μM, 6 μM, and 9 μM for 48 h. The cell apoptotic rate was detected by flow cytometry.

Furthermore, we determined the cell apoptosis rate in the Jeko-1, Rec-1, and Z138 after treatment with BGB-3111 at increasing doses of 0 μM, 3 μM, 6 μM, and 9 μM for 48 h. Figure 4 and Supplementary Figure 2 show that the apoptosis rate of cells increased from 6.35% ± 0.98% to 43.25% ± 4.84%, 8.79% ± 4.13% to 61.62% ± 5.64% and 3.45% ± 0.36% to 28.84% ± 6.23% in the Jeko-1, Rec-1, and Z138 cells after the BGB-3111 treatment (*p* < 0.05), suggesting that BGB-3111 significantly promotes cellular apoptosis in MCL.

### ITK expression regulated by BGB-3111 and another BTK inhibitor, ibrutinib in the T-cells

**Figure 5:**
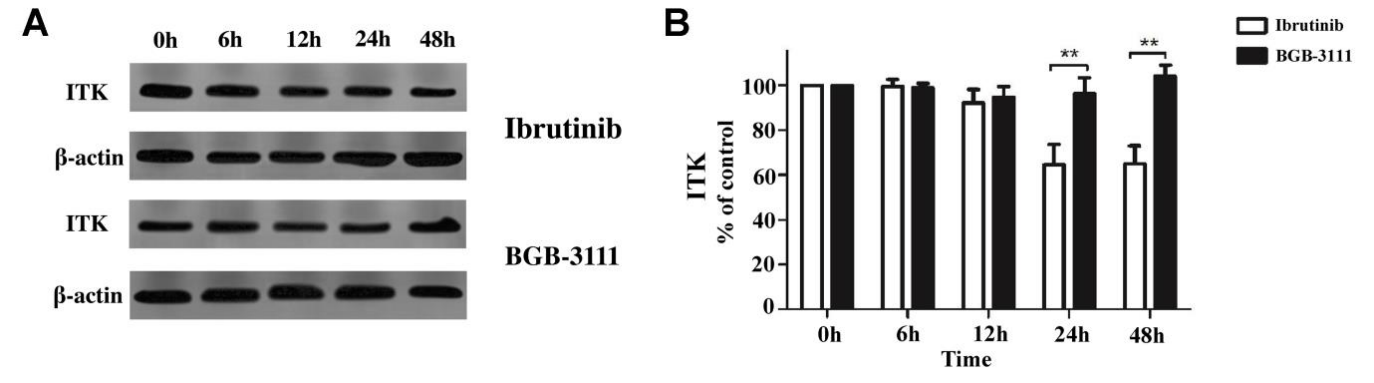
**The ITK expression is regulated by BGB-3111 and ibrutinib in the Jurkat T-cells.** (**A**) The ITK expression in the Jurkat T-cells treated with BGB-3111 and ibrutinib at the same dose of 2 μM for 0–48 h. (**B**) The percentage of inhibition rate of the ITK protein in the Jurkat T-cells treated with BGB-3111 and ibrutinib. ^**^p < 0.01.

Ibrutinib was the first BTK inhibitor approved by the FDA for clinical use. However, its adverse events, especially AF, occur in approximately 5% of MCL patients. A previous study has shown that ibrutinib has off-target effects, involving the Tec family of proteins, such as the inducible T-cell kinase (ITK) in T-cells through PI3K/AKT signaling [22]. To further characterize this effect of both the drugs in T-cells, we measured the ITK protein in stimulated Jurkat T-cells treated with BGB-3111 or ibrutinib at the same dose of 2 μM for 0–48 h. The results showed that after treatment for more than 24 h, the ITK protein was inhibited by ibrutinib, but not with BGB-3111 (Figure 5). These data showed that BGB-3111 is a selective BTK inhibitor and does not inhibit ITK in the T-cells, suggesting that BGB-3111 has the potential to provide better safety in clinical use compared to ibrutinib.

### BTZ enhanced the inhibitory effect mediated by BGB-3111 in the MCL cells

**Figure 6:**
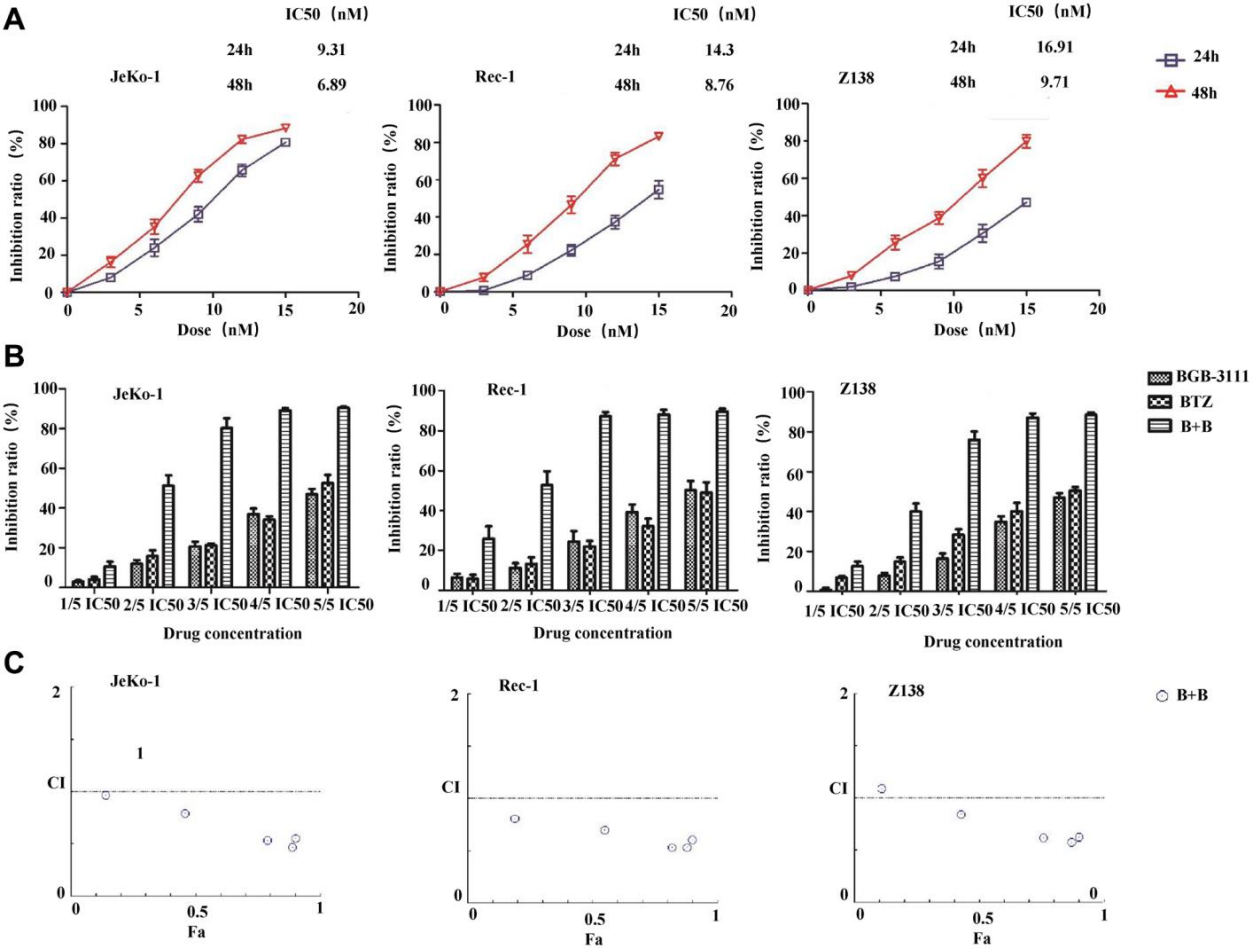
**Low-dose BTZ enhances the inhibitory effect mediated by the low-dose BGB-3111 in the MCL cells.** (**A**) Cell viability in the Jeko-1, Rec-1, and Z138 treated with varying doses of BTZ for 24 or 48 h. (**B**) Cell viability in the Jeko-1, Rec-1, and Z138 treated with single or combination of low-dose BGB-3111 and BTZ for 48 h. (**C**) The combination index of cell proliferation in the Jeko-1, Rec-1, and Z138 treated with low-dose BGB-3111 combined with low-dose BTZ. Results are the mean ± SD of three independent experiments.

We first examined the cytotoxic effects of BTZ in the MCL cells expressing BTK. Results showed that BTZ reduced the cell viability in a dose- and time-dependent manner in the Jeko-1, Rec-1, and Z138 cells (Figure 6A). The mean IC50 values were 9.31 nM, 14.3 nM, and 16.91 nM, respectively after treatment for 24 h, and 6.89 nM, 8.76 nM, and 9.71 nM after treatment for 48 h in the Jeko-1, Rec-1, and Z138 cells. Furthermore, the three MCL cell lines were treated with BGB-3111 and/or BTZ at concentrations that were 20%, 40%, 60%, 80%, and 100% of the IC50 for 48 h. Figure 6B reveals that the combination of low-dose BGB-3111 with low-dose BTZ synergistically increased the inhibition rate in Jeko-1, Rec-1, and Z138 cells compared to the treatment with a single agent. Figure 6C shows that the combination treatment synergistically inhibited cell proliferation in all the three MCL cell lines (CI<1). These data revealed that a low dose of BTZ enhanced the inhibitory effect mediated by the low-dose BGB-3111 in the MCL cells, suggesting that it is a potential therapeutic strategy to combine the low doses of both the drugs to gain effective anti-cancer effects and better safety for MCL.

### BTZ enhanced cell apoptosis and cell-cycle arrest induced by BGB-3111 in the MCL cells

**Figure 7:**
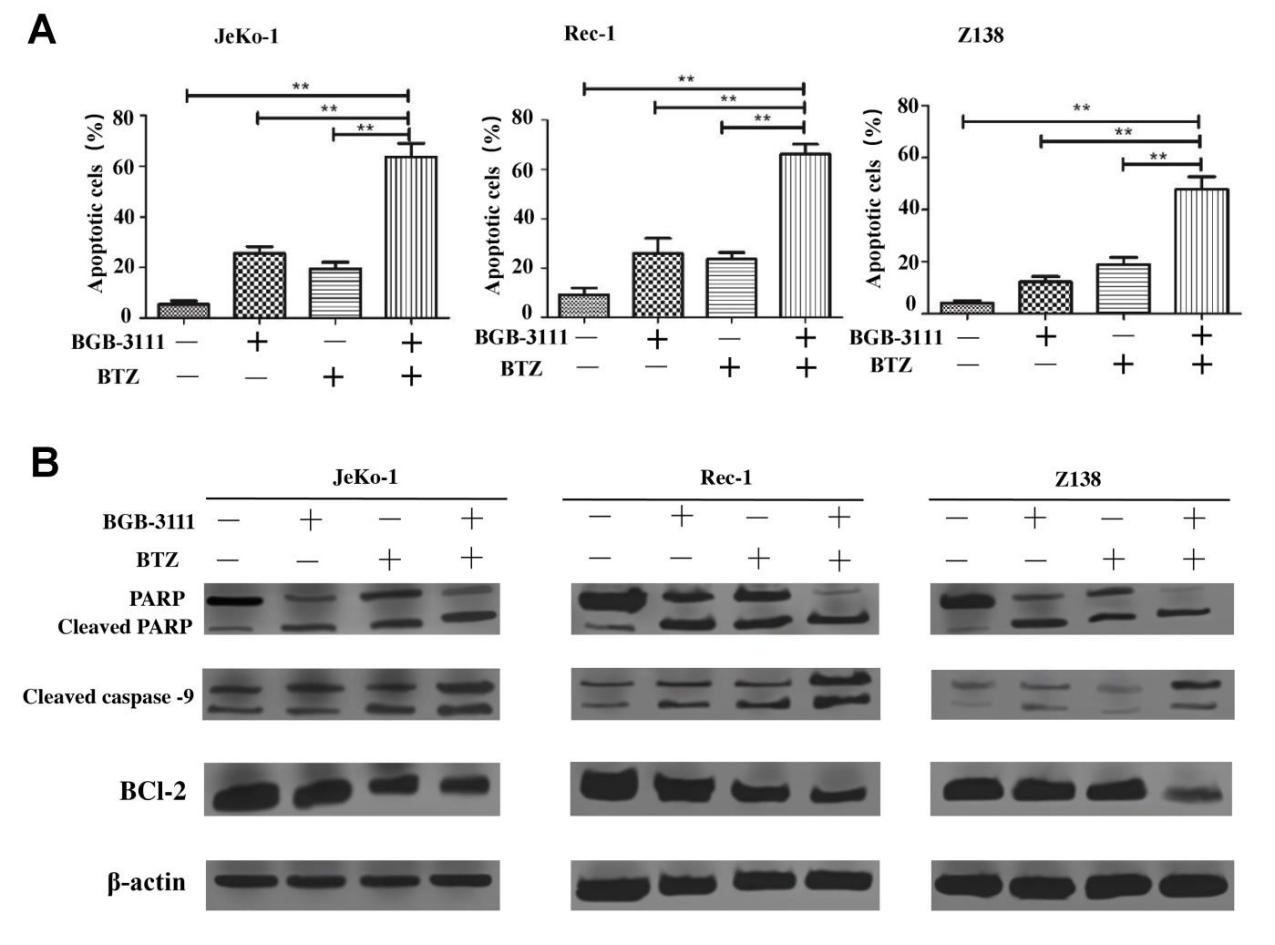
**Low-dose BTZ enhances the cell apoptotic effect induced by low-dose BGB-3111 in the MCL cells.** (**A**) Cell apoptotic distribution of the Jeko-1, Rec-1, and Z138 cells after being treated with low-dose BGB-3111, low-dose BTZ, and their combination for 48 h. (**B**) The cell apoptotic rate was detected by flow cytometry. ^**^p < 0.01. The expression levels of the apoptosis-related proteins in Jeko-1, Rec-1, and Z138 cells after treating with low-dose BGB-3111, low-dose BTZ, and their combination for 48 h.

According to the above results, the inhibitory effect of 20% of the IC50 for both drugs is less limiting; 40% of the IC50 for BGB-3111 combined with 40% of the IC50 for BTZ could almost inhibit half of the cell proliferation, and we would like to use a low concentration to achieve a good inhibitory effect on the tumor cell growth. Therefore, we further selected the low dose of BGB-3111 (40% of the IC50) and BTZ (40% of the IC50) to investigate the cell cycle arrest and cell apoptosis in the MCL cells expressing BTK. Figure 7A and Supplementary Figure 3 show that the combination of low-dose BGB-3111 with low-dose BTZ resulted in a significant increase in cell apoptosis compared to a single agent in the Jeko-1, Rec-1, and Z138 cells after treatment for 48 h. The apoptotic rates were 24.91% ± 3.47% and 17.63% ± 4.34% in Jeko-1 cells for low-dose BGB-3111 and low-dose BTZ, respectively; however, it significantly increased to 63.19% ± 6.65% for the combinatorial treatment of both low-dose drugs. The apoptotic rates were 23.64% ± 7.86% and 22.25% ± 3.39% in Rec-1 cells for low-dose BGB-3111 and low-dose BTZ, respectively; however, it significantly increased to 64.89% ± 5.47% for the combination treatment with both the low-dose drugs. The apoptotic rate was 12.56% ± 2.02% and 19.80% ± 3.42% in Z138 cells for low-dose BGB-3111 and low-dose BTZ, respectively; however, it significantly increased to 48.42% ± 5.88% for the combination treatment with both low-dose drugs. At the same time, low-dose BTZ enhanced the cell cycle arrest induced by low-dose BGB-3111. The percentage of G1/G0 phase cells was 48.66% ± 1.98% and 37.51% ± 5.12% in the Jeko-1 cells for low-dose BGB-3111 and low-dose BTZ, respectively; however, it significantly increased to 77.48% ± 3.25% for the combinatorial treatment of both the low-dose drugs. The percentage of G1/G0 phase cells was 60.10% ± 5.12% and 40.69% ± 3.28% in the Rec-1 cells for low-dose BGB-3111 and low-dose BTZ, respectively; however, it significantly increased to 80.74% ± 2.22% for the combinatorial treatment of both the low-dose drugs. The percentage of G1/G0 phase cells was 46.92% ± 1.21% and 30.75% ± 3.01% in Z138 cells for low-dose BGB-3111 and low-dose BTZ, respectively; however, it significantly increased to 75.22% ± 2.67% for the combinatorial treatment of both the low-dose drugs (Supplementary Figure 4). These results suggest that low-dose BTZ enhanced the cell apoptosis and cell-cycle arrest induced by low-dose BGB-3111 in the MCL cells.

### BTZ enhanced the anti-cancer effect of BGB-3111 by regulating the apoptosis-related proteins

To understand the underlying mechanism of the anti-cancer effect, the expression levels of the apoptosis-related proteins, including PARP, caspase-9, and Bcl-2, were detected in the Jeko-1, Rec-1, and Z138 cells after treatment with low-dose BGB-3111 (40% of the IC50) and BTZ (40% of the IC50). Figure 7B shows that the combination of low-dose BGB-3111 with low-dose BTZ enhanced the pro-apoptotic effect compared to a single agent treatment, increased cleaved PARP and caspase-9 expression, and inhibited Bcl-2 expression. These data indicate that the expression of PARP, cleaved caspase-9 and Bcl-2 were significantly regulated in combination treatment, suggesting that these apoptosis-related proteins were involved in the anti-cancer process.

### BTZ enhanced the anti-cancer effect of BGB-3111 by synergistically suppressing the NF-κB pathway

**Figure 8:**
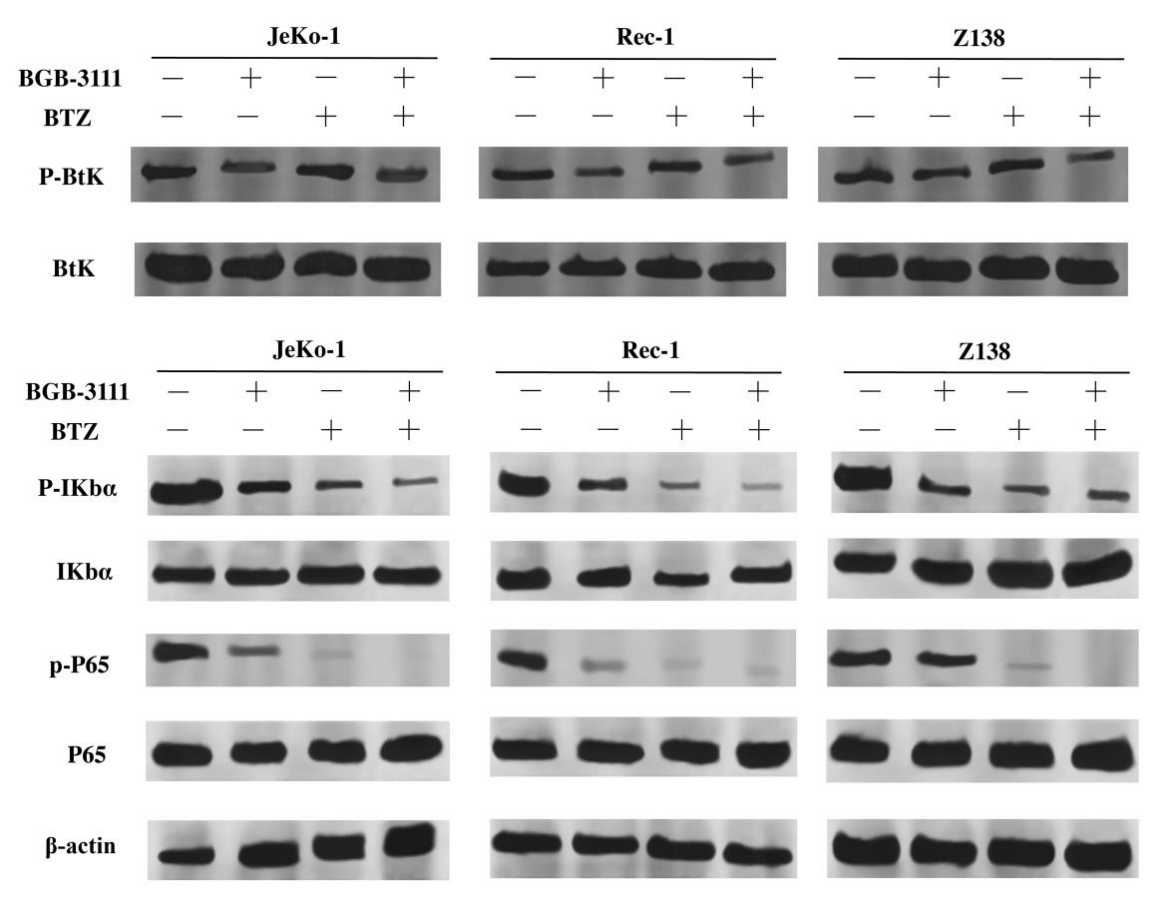
Expression levels of BTK, IκBα, P65 and their phosphorylation proteins in the Jeko-1, Rec-1, and Z138 cells after treating with low-dose BGB-3111, low-dose BTZ, and their combination for 48 h.

The NF-κB signaling pathway is downregulated by BTK as well as BTZ. Next, we investigated whether the expression of key proteins involved in NF-κB signaling was regulated in the Jeko-1, Rec-1, and Z138 cells after treatment with low-dose BGB-3111 (40% of the IC50) and BTZ (40% of the IC50). The results showed that the expression of the p-BTK protein was downregulated in these cells after treatment with low-dose BGB-3111, but not BTZ. The expression of p-IκBα and p-P65 proteins were decreased in these cells after treatment with low-dose BGB-3111 or low-dose BTZ, but the combination strongly suppressed these protein expressions compared to the single-agent (Figure 8).

## DISCUSSION

MCL is a relatively rare subtype of B-cell lymphoma with a poor prognosis. Ibrutinib, a first-generation BTK inhibitor, has shown promising efficacy in CLL/SLL, MCL, and WM [48–50]. However, it poses adverse effects usually appearing as off-target effects on the other kinases, including Tec family proteins. A novel and next-generation BTK inhibitor, BGB-3111, has been shown to be well-tolerated and highly active in CLL/SLL and WM in preclinical studies [32, 35]. Herein, we found that BGB-3111 strongly inhibited cell proliferation, induced cell cycle arrest in the G1/G0-phase, and promoted cell apoptosis in the MCL cells expressing BTK, and showed a restricted off-target activity on ITK protein compared to ibrutinib. In combination with BTZ, low-dose BGB-3111 synergistically enhanced the anti-cancer effect by increasing cell-cycle arrest, regulating apoptosis-related proteins, and inhibiting the NF-κB pathway.

BTK is a key kinase in the BCR signaling pathway and shows persistent activation in MCL [51]. In this study, we found that 59.4% of MCL patients expressed the BTK protein, which is consistent with a previous report [52]. This suggests that targeting BTK is a promising treatment strategy for patients with MCL [53, 54]. Cell proliferation is closely related to the cell cycle and apoptosis, and their dysregulations are the main characteristics of MCL [55]. Our results revealed that BGB-3111 decreased cell proliferation, induced cell apoptosis, and arrested the cell cycle in the G1/G0-phase in MCL-expressing BTK.

BGB-3111 and ibrutinib have the same antineoplastic mechanism that binds to the Cys-481 residue in BTK. BTK belongs to a member of the Tec family of tyrosine kinases but is specifically expressed in the B-cells. Other Tec family members, such as ITKs, are expressed in the T-cells. Phosphorylation of ITK can activate its downstream signaling pathways [56] and lead to some toxicities, such as AF [57]. Our results showed that BGB-3111 had a restricted off-target activity on ITK in T-cells compared to ibrutinib, suggesting that BGB-3111 may have better safety. Moreover, we found that the combination of low-dose BGB-3111 with low-dose BTZ significantly enhanced the anti-cancer effects by increasing the expression of cleaved PARP and caspase-9 and inhibiting Bcl-2 expression and further affecting the NF-κB pathway. The NF-κB signaling is constitutively activated in the MCL cells, which involves tumorigenesis by promoting cellular survival signals [58]. In this study, low-dose BGB-3111 treatment decreased the expression of BTK phosphorylation, whereas BTZ treatment did not change BTK phosphorylation. However, both low-dose BGB-3111 and low-dose BTZ alone decreased the phosphorylation of IκB-α and P65 phosphorylation in the MCL cells. The combination of low-dose BGB-3111 with low-dose BTZ enhanced the anti-cancer effects through the NF-κB pathway. However, this was only a preliminary finding of the synergistic effect of BGB-3111 and BTZ, and further studies are needed to explore the detailed mechanism and the effect on the resistance of the MCL cell lines. Our data provide a potential therapeutic strategy for the combinatorial treatment of the low dose of both the drugs to gain strong anti-cancer effects and better safety for the patients with MCL.

In conclusion, BTK is highly expressed in the tissues of MCL patients. BGB-3111 is a potentially novel and effective BTK inhibitor for the treatment of MCL with a higher BTK specificity compared to ibrutinib. Low-dose BTZ might further enhance the anti-cancer effect of low-dose BGB-3111 in MCL expressing BTK, suggesting that the combination of low-dose BGB-3111 and low-dose BTZ is a potential and effective therapeutic strategy for MCL patients.

## MATERIALS AND METHODS

### Patient samples

We obtained 32 formalin-fixed paraffin-embedded (FFPE) tissues from the MCL patients at the Tianjin Medical University Cancer Institute and Hospital (TMUCIH). Each specimen was reviewed by two experienced hematopathologists for diagnostic confirmation. In addition, 10 specimens of the normal lymph gland tissue obtained from patients with reactive hyperplasia of the lymph nodes in TMUCIH were used as controls. The study was approved by the Clinical Research Ethics Board of TMUCIH, and written informed consent was provided by all the participating patients. This study was conducted in accordance with the 1964 Declaration of Helsinki.

### Immunohistochemistry and scoring of BTK

The immunohistochemical staining was performed to quantify the BTK proteins. Briefly, the FFPE slides were dewaxed, rehydrated, and antigen-retrieved (Tris–EDTA buffer, pH 9.0). Next, they were blocked with 10% goat serum for 30 min at 37°C before incubating the sample in the BTK primary antibody (1:200, Cell Signaling Technology Inc., MA) overnight at 4°C. They were then incubated with a secondary antibody for 60 min at 37°C. The chromagen 3, 3′-diaminobenzidine (DAKO, Carpenteria, CA) was applied for 5 min, and the slides were counterstained with hematoxylin and then stored at 4°C before imaging under a microscope. The positive percentage and staining intensity of the tumor cells were evaluated for each slide. Briefly, the positive percentage was classified into 0–3 scores (0, 0–5% positivity; 1, 5–25% positivity; 2, 25–50% positivity; and 3, ≥50% positivity), and the staining intensity was also categorized into 0–3 scores (0, absent; 1, weak; 2, moderate; and 3, strong). The expression of BTK was evaluated by two experienced hematopathologists who were blinded to the sample identities. The semiquantitative H-scores were calculated by multiplying the percentage score by the intensity score. The high BTK expression was defined as H-scores ≥ 6, and the low BTK expression was defined as H-scores < 6.

### Cell lines and reagents

Human MCL cell lines including Jeko-1, Rec-1, Z138, Grant 519, and Mino, and human T-cell line, Jurkat were provided by Dr. Kai Fu (University of Nebraska Medical Center, NE, USA). These cells were maintained in the RPMI 1640 media supplemented with 10% fetal bovine serum (FBS, Thermo Fisher Scientific, MA, USA) and 1% penicillin/streptomycin was added, and cultured at 37°C with 5% CO_2_. The BGB-3111 was supplied as a gift by BeiGene Ltd. (Beijing, China). BTZ and ibrutinib were purchased from Selleck Chemicals (Houston, TX, USA).

### Cell viability assay

The cells were treated with various doses of BGB-3111 or/and BTZ, and then cell viability was determined after 48 h or 72 h incubation using a CellTiter 96 Aqueous One Solution Cell Proliferation Assay (Promega, Madison, WI, USA, Cat. #G3580) according to the manufacturer’s protocol. The IC50 (50% inhibitory concentration) values were calculated using GraphPad Prism software (version 5.0). The data were confirmed by performing at least three independent experiments.

### Cell cycle assay

The cells were treated with BGB-3111 or/and BTZ for 48 h, washed, resuspended in cold PBS, and fixed by dropwise addition of ice-cold absolute ethanol to a final concentration of 70%. The fixed cells were stored at 4°C overnight, pelleted, and incubated with RNase A (Solarbio, China, final concentration of 0.2 mg/ml), and propidium iodide (Sigma-Aldrich, final concentration was 50 μg/ml) for 30 min at 37°C in the dark. The cell cycle was analyzed on a FACSCelesta flow cytometer (BD Bioscience, CA, USA) using ModFit LT 4.1 software.

### Cell apoptosis assay

The cells were treated with BGB-3111 and/or BTZ for 48 h and then rinsed with cold PBS. After centrifugation at 300 × *g* for 5 min, cells were resuspended in 100 μl binding buffer (BD Biosciences, CA, USA), and then 5 μl of Annexin V-FITC and 5 μl of PI were added following the apoptosis detection kit manufacturer’s protocol (BD Biosciences, San Diego, CA). The samples were analyzed by FACSCelesta flow cytometry after incubation for 15 min in the dark at room temperature (RT). The results were analyzed using FlowJo V10 software.

### Synergy evaluation of the anti-cancer effect in combinatorial treatment

The cells were treated with BGB-3111 and/or BTZ at concentrations that were 20%, 40%, 60%, 80%, and 100% of the IC50 for 48 h. Cell viability was determined as described above. The effects of the combined treatment were calculated by the CompuSyn software using the Chou–Talalay combination index (CI) and isobologram methods according to the median-effect principle. The combination effect was designated as synergism (CI < 1.0), addition (CI = 1.0), or antagonism (CI > 1.0).

### Western immunoblotting

The treated cells were harvested in the RIPA lysis buffer containing 1 mM PMSF and phosphatase inhibitor cocktail. The whole-cell lysates were centrifuged at 12,000 × *g* at 4°C for 10 min, and the total protein was quantitated using the BCA method (Thermo Fisher Scientific, MA, USA). A 20–50 μg of Total protein was separated by the 10% SDS-PAGE gel electrophoresis and subsequently transferred to the PVDF membranes (Roche, UK) at 260 mA for 2 h. Then, the blot was blocked in 5% bovine serum albumin for 1 h at RT and incubated with the specific primary antibodies at 4°C overnight. After washing three times in Tris-buffered saline–Tween for 10 min, the blots were probed with 1:2000 dilution of goat anti-rabbit IgG or anti-mouse IgG horseradish peroxidase-conjugated secondary antibodies (Sigma-Aldrich, MO, USA) for 1 h at RT. The signals were detected using the chemiluminescence reagent Immobilon (Merck Millipore, Darmstadt, Germany). The signal intensity was obtained using the Image Studio Lite software. Antibodies, including the rabbit anti-human BTK, rabbit anti-human phospho-BTK, mouse anti-human cleaved caspase-9, mouse anti-human PARP, rabbit anti-human IκBα, rabbit anti-human phospho-IκBα, mouse anti-human P65, and rabbit anti-human phospho-P65, were obtained from the Cell Signaling Technology Inc. (Beverly, MA, USA). The rabbit anti-human Bcl-2 antibody was purchased from ProteinTech Group, Inc. (Hubei, China). The mouse monoclonal anti-β-actin was supplied by Sigma-Aldrich and used as an endogenous protein for normalization.

### Statistical analysis

All the assays were presented as mean ± standard deviation (SD) and were examined for significant differences using the Student’s *t-*test. The differences were analyzed by the one-way analysis of variance using the IBM SPSS Statistics 20 software. Statistical significance was set at *P* < 0.05.

## Supplementary Materials

Supplementary Figures

Supplementary Table 1
