# Supplementary material for: Bortezomib enhances the anti-cancer effect of the novel Bruton’s tyrosine kinase inhibitor (BGB-3111) in mantle cell lymphoma expressing BTK
Source: Aging (Albany NY). 2021 Sep 10;13(17):21102–21. doi: 10.18632/aging.203314 (PMC8457562; doi:10.18632/aging.203314)
Supplement: Supplementary Figures [file aging-13-203314-s001.pdf]

SUPPLEMENTARY FIGURES

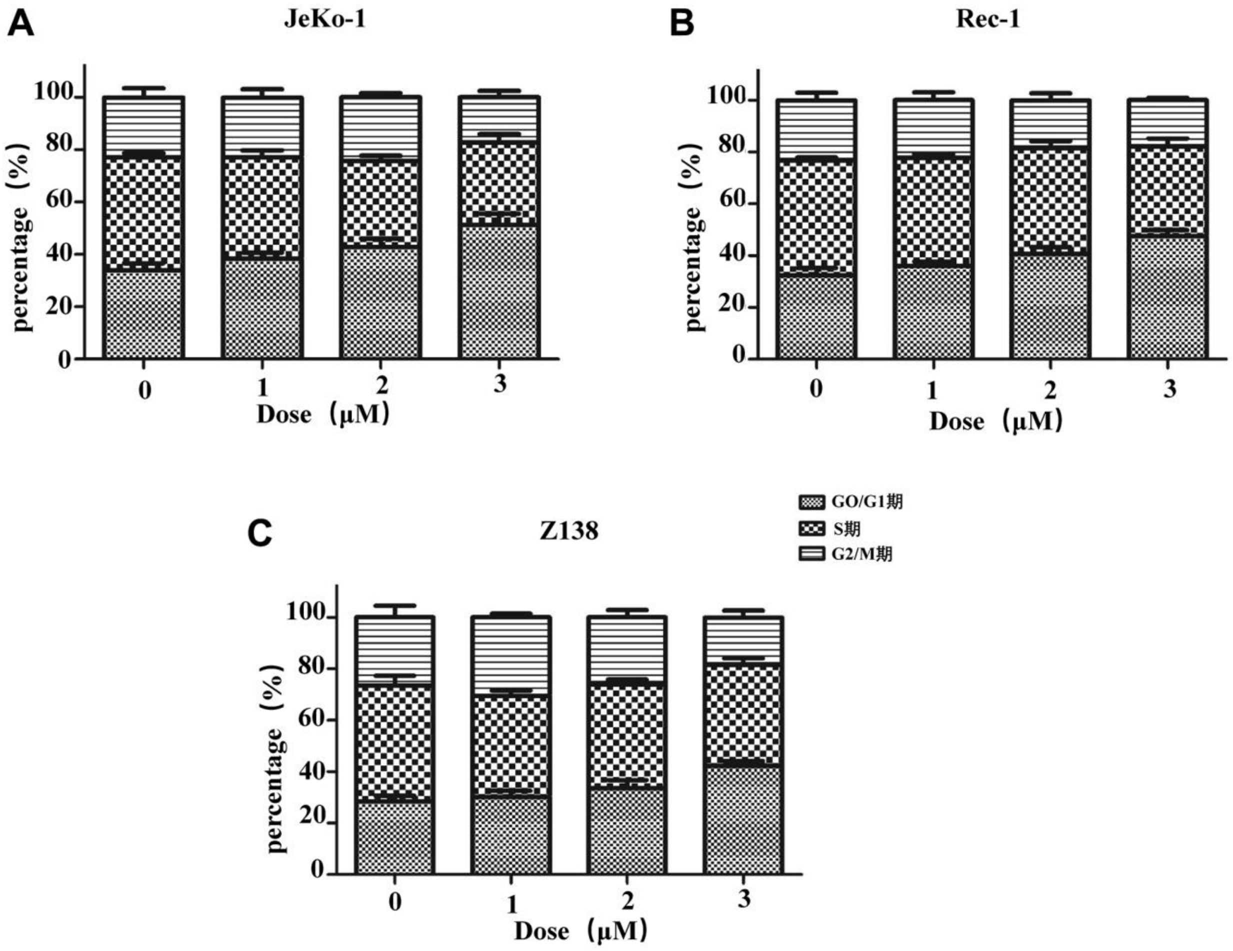

Supplementary Figure 1. Cell-cycle diagrams of Jeko-1 (**A**) Rec-1 (**B**) and Z138 (**C**) cells after treating with various concentrations of BGB-3111 for 48 h. The cell percentage of the cell cycle was detected by flow cytometry.

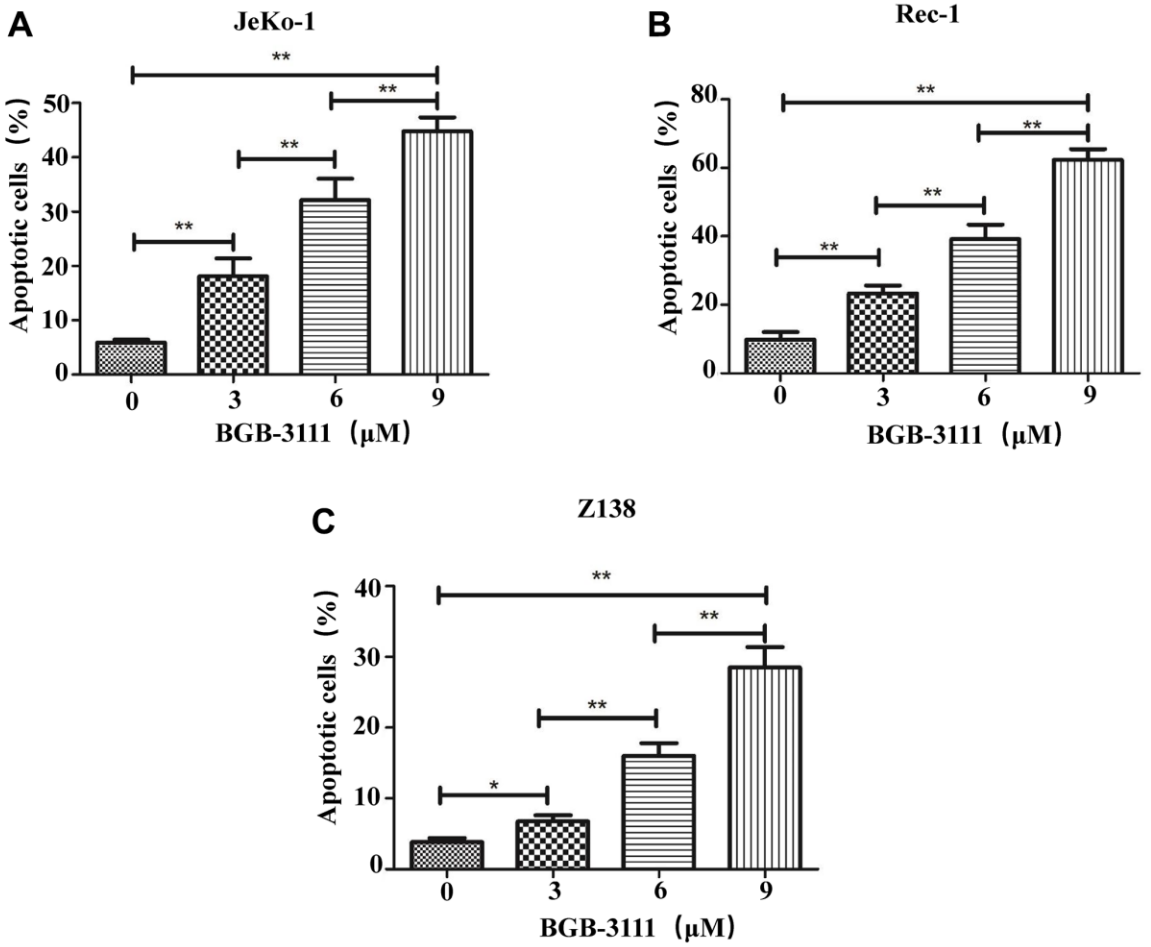

Supplementary Figure 2. Cell apoptotic distribution of Jeko-1 (**A**) Rec-1 (**B**) and Z138 (**C**) cells after treating with various concentrations of BGB-3111 for 48 h. Cell apoptotic rate was detected by flow cytometric analysis. $^{*}p < 0.05$, $^{**}p < 0.01$.

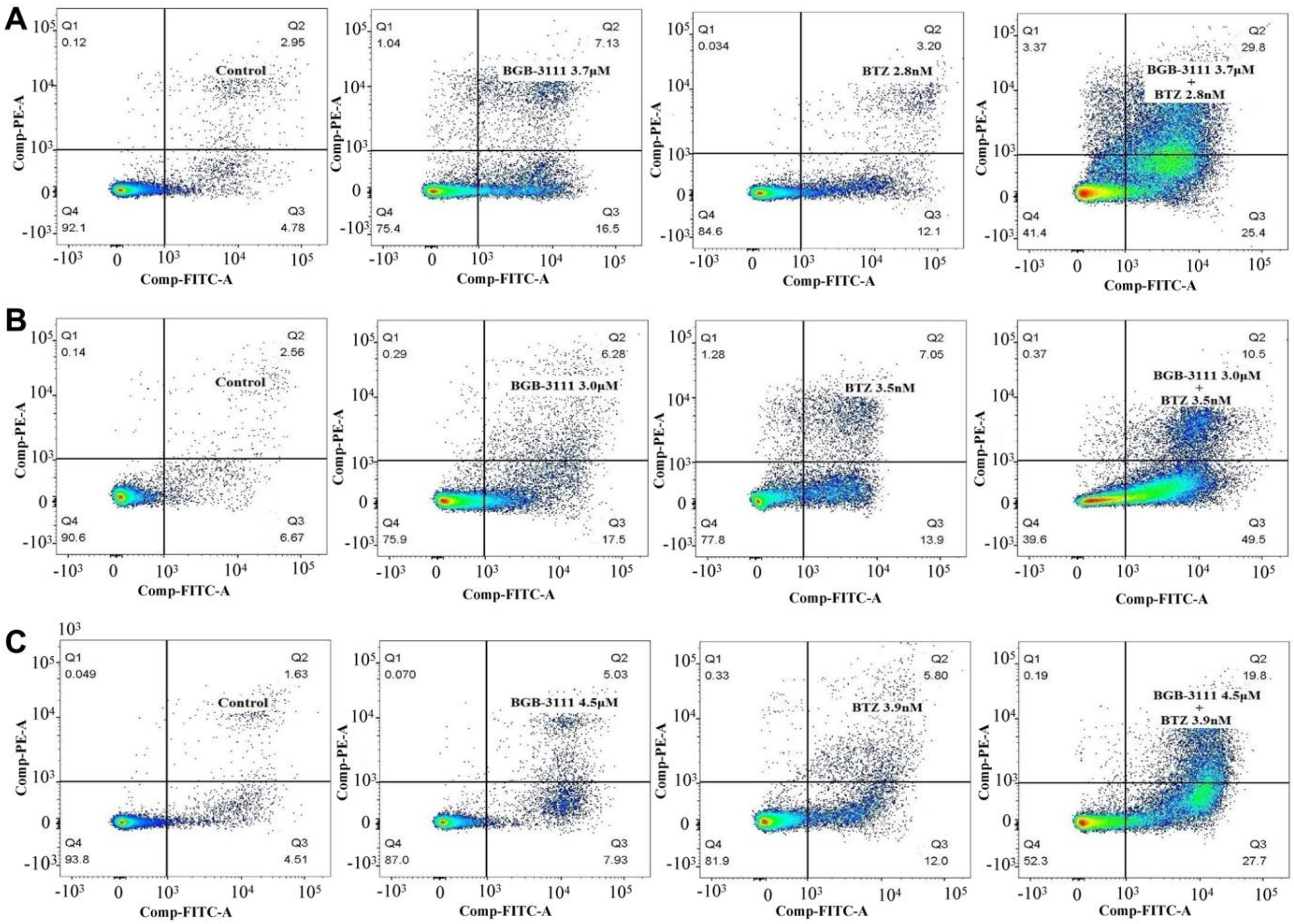

Supplementary Figure 3. Cell apoptosis in Jeko-1 (**A**) Rec-1 (**B**) and Z138 (**C**) cells after treating with low-dose BGB-3111, low-dose BTZ, and their combination for 48 h. Cell apoptotic rate was detected by flow cytometry.

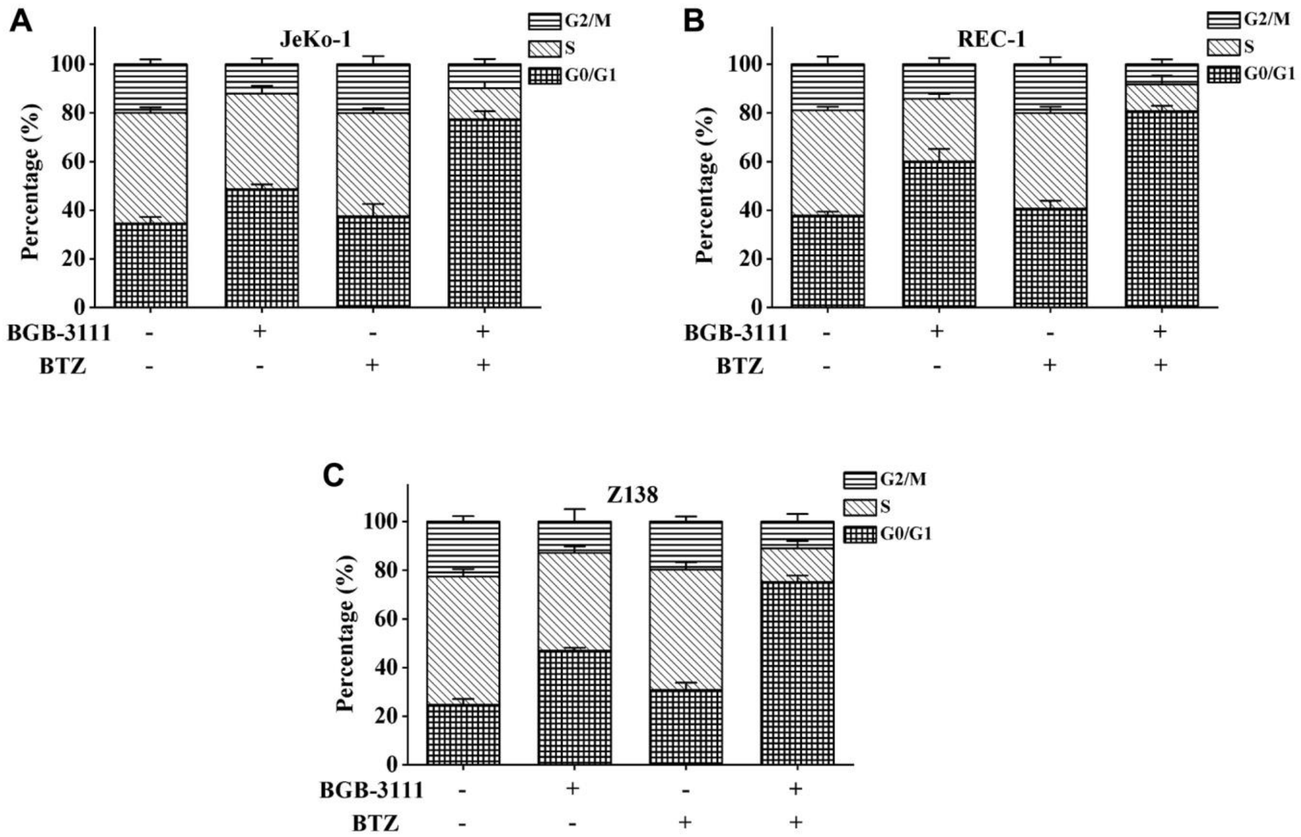

Supplementary Figure 4. Cell-cycle diagrams of Jeko-1 (**A**) Rec-1 (**B**) and Z138 (**C**) cells after treating with various concentrations of BGB-3111 and BTZ for 48 h. The cell percentage of the cell cycle was detected by flow cytometry.
