# Supplementary material for: Bortezomib enhances the anti-cancer effect of the novel Bruton’s tyrosine kinase inhibitor (BGB-3111) in mantle cell lymphoma expressing BTK
Source: Aging (Albany NY). 2021 Sep 10;13(17):21102–21. doi: 10.18632/aging.203314 (PMC8457562; doi:10.18632/aging.203314)
Supplement: Supplementary Table 1 [file aging-13-203314-s002.pdf]

SUPPLEMENTARY TABLE

Supplementary Table 1. Association between BTK expression and clinicopathologic parameters.

Characteristics	Total (*n* = 32)	BTK expression		χ^2	*P* value
		High (*n* = 19)	Low (*n* = 13)		
**Gender**				0.076	0.545
Male	23 (72%)	14 (73.7%)	9 (69.2%)		
Female	9 (28%)	5 (26.3)	4 (30.8%)		
**Age**				1.943	0.153
<65	20 (63%)	10 (52.6%)	10 (76.9%)		
≥65	12 (37%)	9 (47.4%)	3 (23.1%)		
**ECOG score**				4.055	0.058
0–1	27 (84%)	14 (73.7%)	13 (100%)		
2–4	5 (16%)	5 (26.3%)	0 (0%)		
**B symptoms**				0.162	0.487
No	21 (66%)	13 (68.4%)	8 (61.5%)		
Yes	11 (34%)	6 (31.6%)	5 (38.5%)		
Stage				4.838	0.051
I-II	3 (9%)	0 (0%)	3 (23.1%)		
III-IV	29 (91%)	19 (100%)	10 (76.9%)		
**BM involvement**				2.496	0.114
No	12 (37%)	5 (26.3%)	7 (53.8%)		
Yes	20 (63%)	14 (73.7%）	6 (46.2%)		
**WBC/mm**				0．173	0.327
<10,000	21 (66%)	12 (63.2%)	9 (69.2%)		
≥10,000	11 (34%)	7 (36.8%)	4 (30.8%)		
**β2-MG**				0.275	0.521
≤3 mg/L	18 (56%)	8 (47%)	10 (67%)		
>3 mg/L	14 (44%)	9 (53%)	5 (33%)		
**LDH/(IU/L)**				0.13	0.5
<250	16 (50%)	9 (47.4%)	7 (53.8%)		
≥250	16 (50%)	10 (52.6%)	6 (46.2%)		
**Ki-67**				5.783	0.019*
≤30%	19 (59%)	8 (42.1%)	11 (84.6%)		
>30%	13 (41%)	11 (57.9%)	2 (15.4%)		
**MIPI score**				4.522	0.038*
Low-Middle risk (<6)	23 (72%)	11 (57.9%)	12 (92.3%)		
Middle-High risk (≥6)	9 (28%)	8 (42.1%)	1 (7.7%)		
